# CHD1L Protein is overexpressed in human ovarian carcinomas and is a novel predictive biomarker for patients survival

**DOI:** 10.1186/1471-2407-12-437

**Published:** 2012-09-29

**Authors:** Wei-Peng He, Juan Zhou, Mu-Yan Cai, Xiang-Shen Xiao, Yi-Ji Liao, Hsiang-Fu Kung, Xin-Yuan Guan, Dan Xie, Guo-Fen Yang

**Affiliations:** 1 Department of Gynecology, the First Affiliated Hospital, Sun Yat-Sen University, No. 78, Zhongshan Road II, 510080, Guangzhou, China; 2 State Key Laboratory of Oncology in South China, Cancer Center, Sun Yat-Sen University, No. 651, Dongfeng Road East, 510060, Guangzhou, China; 3 Department of Oncology, the University of Hong Kong, Hong Kong, China

## Abstract

**Background:**

Our recent studies suggested that **t**he chromodomain helicase DNA binding protein 1-like (CHD1L) gene plays an oncogenic role in human hepatocellular carcinoma. However, the status of CHD1L protein expression in ovarian cancer and its clinical/prognostic significance are obscure.

**Methods:**

In this study, immunohistochemistry (IHC) for CHD1L was performed on a tissue microarray (TMA) containing 102 primary ovarian carcinomas and 44 metastatic lesions (omental metastasis). Receiver-operator curve (ROC) analysis was used to evaluate patients’ survival status.

**Results:**

There is an augmented tendency of CHD1L expression in ovarian carcinoma metastasis than in primary lesions (*P*<0.05). A significant association was found between positive expression of CHD1L and tumors histological type (*P* <0.05). By univariate survival analysis of the ovarian carcinoma cohorts, positive expression of CHD1L was significantly correlated with shortened patient survival (mean 66.7 months versus 97.4 months, *P*<0.05). Moreover, CHD1L expression was evaluated to be a significant and independent prognostic factor in multivariate analysis (*P*<0.05).

**Conclusions:**

These findings provide evidence that positive expression of CHD1L protein is significantly correlated with the metastasis proceeding of ovarian carcinoma, and CHD1L protein expression, as examined by IHC, may act as a novel prognostic biomarker for patients with ovarian carcinoma.

## Background

Ovarian cancer, the leading cause of death from a gynecological malignancy, has increasing incidence recently in China and Singapore [1]. The onset age for ovarian cancer is about 45 years old [2]. On account of the absence of early diagnosis methods and the absence of characteristic symptoms, the majority of ovarian cancer patients (70%) were diagnosed at FIGO III/IV, and had a unfavorable prognosis with a 5-year survival rate of <20% [3]. Recently, ovarian cancer was considered as a more complicated genetic change and multiple-step patho-physiological change [4]. Clinically, most ovarian cancers are ovarian carcinoma. It is prevalent to discovery a tumor marker for the diagnosis and prognosis of ovarian carcinoma, as well as developing targeted therapy, predicting the survival, and predicting relapse.

Recently, we reported that the chromodomain helicase DNA binding protein 1-like (CHD1L) gene is a putative oncogene mapped to chromosome 1q21 and it is associated with hepatocellular carcinoma (HCC) tumorigenesis [5]. CHD1L belongs to SNF-2family, which contains a domain (approximately 400 amino acids) with highly conserved helicase motifs [5]. The full-length mRNA (2.98kb) of CHD1L encodes an 897aa protein. Most SNF2-like proteins participate in various nuclear activities, such as transcriptional activation or repression, DNA repair and recombination [6,7]. In HCC patients, over 50% cases have overexpression of CHD1L, which is associated with the carcinoma progression and metastasis and predict the poor prognosis of the survival status [8]. To date, despite these facts, the status of CHD1L protein in ovarian carcinoma tissue and its prognostic significance has not been elucidated.

In this study, therefore, we examined the expression status of CHD1L protein by IHC in a cohort of ovarian carcinoma tissues (including 102 primary lesions and 44 corresponding metastatic lesions). The clinical/prognostic significance of CHD1L expression in our ovarian carcinoma cohort was assessed.

## Methods

### Patients and tissue specimens

In this study, paraffin-embedded tissue samples from 102 patients with ovarian carcinoma and corresponding 44 cases metastasis were obtained from the archives of Department of Pathology, the First Affiliated Hospital, Sun Yat-Sen University, Guangzhou, China, between 1996 and 2008. The patients selected were based on availability of resection tissue, follow-up data, and had not accepted preoperative radiation or chemotherapy. Patients died from unknown causes or emergency were excluded from this study. The age of the 102 patients with ovarian carcinoma ranged from 18 to 86 years (mean, 51.0 years) and their clinico-pathological characteristics are described in Table 1. Prior patient’s informed consent and the Institute Research Medical Ethics Committee of Sun Yat-Sen University granted approval for this study.

**Table 1:** Association of CHD1L protein expression with clinic-pathological parameter

		**CHD1L protein expression**		
**Variable**	**Allcase**	**negative**	**positive**	***P *Value**^a^
Age at surgery (years)				0.168
≤ 51 ^b^	54	23(52.6%)	31(57.4%)	
> 51	48	27(56.2%)	21(43.8%)	
Histological type				
Serous	72	35(48.6%)	37(51.4%)	0.029
Mucinous	11	9(81.8%)	2(18.2%)	
Others^c^	19	6(32.6%)	13(68.4%)	
Histologicalgrade(Silveberg)				0.745
G1	16	9(56.2%)	7(43.8%)	
G2	61	30(48.2%)	31(50.8%)	
G3	25	11(44.0%)	14(56.0%)	
FIGO stage				0.354
I	19	8(42.1%)	11(57.9%)	
II	10	7(70.0%)	3(30.0%)	
III	58	26(44.8%)	32(55.2%)	
IV	15	9(60.0%)	6(40.0%)	
Residual tumor				0.469
None	32	15(46.9%)	17(53.1%)	
<=2cm	35	15(42.9%)	20(57.1%)	
>2cm	35	20(47.1%)	15(42.9%)	

^a^Chi-square test.

^b^ Mean age.

^c^Endometrioid, Clear cell and Undifferentiated types.

### Construction of tissue microarrays (TMA)

The tissue microarray was constructed as previously described [9-11]. The formalin-fixed, paraffin-embedded tissue blocks and the corresponding histological HE-stained slides were overlaid for tissue TMA sampling. Our senior pathologist reviewed the slides and marked the representative areas of tumor tissues. We used a tissue arraying instrument (Beecher Instruments, Silver Spring, MD, USA) to punch triplicate 0.6-mm-diameter cylinders of tissue from selected cancer areas of individual donor tissue block and re-embed into a recipient paraffin block at a predefined position. The TMA blocks included 102 ovarian carcinomas and 44 corresponding metastasis lesions. Subsequently, multiple sections (5-um thick) were cut from the TMA tissue array.

### Immunohistochemistry (IHC)

The immunohistochemical study of CHD1L was performed using a standard streptavidin-peroxidase method demonstrated previously [12]. The endogenous peroxidase activity was blocked with 3% H_2_O_2_ for 10 minutes. For the antigen retrieval, slides were immersed in 10 mM citrate buffer (pH 6.0) and boiled for 15 minutes in a microwave oven. Non-specific binding was blocked by 5% normal goat serum for 10 minutes. The slides were incubated with a 1:100 dilution of monoclonal antibody against CHD1L (Abcam) at 4°C overnight in a moist chamber. The slides were sequentially incubated with biotinylated goat anti-mouse IgG (1:100 dilution; Santa Cruz Biotechnology) and then streptavidin-peroxidase conjugate, each for 30 minutes at room temperature. Isotope-matched human IgG was used in each case as a negative control. Finally, the 3, 5-diaminobenzidine (DAB) Substrate Kit (Dako) was used for color development followed by Mayer hematoxylin counterstaining.

For the evaluation of CHD1L staining, a semi-quantitative scoring criterion was used, in which both staining intensity and positive cells percentage were contained [12,13]. A staining index (with values from 0 to 12) was obtained as the intensity of CHDIL staining (0=negative, 1=weakly positive, 2=positive, 3=strongly positive) times the proportion of immunopositive tumor cells (0%=0;<10%=1;10%≤ to<50%=2;50%≤to <75%=3;≥75%=4 ). All histological evaluations were carried out in a double-blind manner by two expert pathologists (M-YC and DX).

### Statistical analyses

Statistical analysis was executed with the SPSS statistical software package (SPSS Standard version 13.0, SPSS Inc.). We assess the association of CHD1L protein expression with clinico-pathological features by the Chi-square test or Fishers’ exact test. Wilcoxon Signed Ranks Test is used to compare the CHD1L protein expression between the primary ovarian carcinomas and metastasis. Multivariate survival analysis was performed on all factors which have been proved to be significant on univariate analysis by the Cox regression model. For univariate survival analysis, Kaplan–Meier analysis is used. We utilized Log rank test to compare different survival curves. ROC analysis is used to compare the clinico-pathological features for estimation of the survival prediction. *P* value of <0.05 was considered statistically significant.

## Results

### CHD1L protein expression in primary ovarian carcinomas and corresponding metastatic lesions

**Figure 1:**
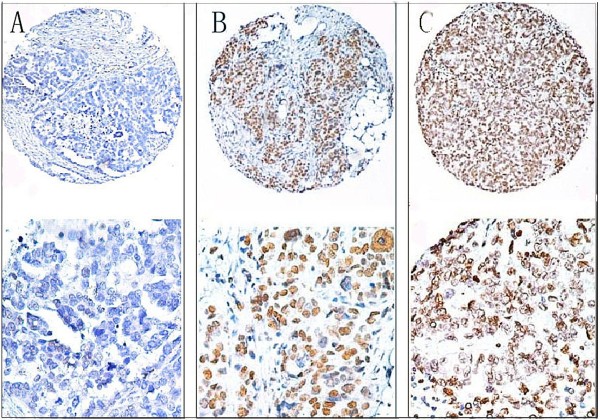
**The protein expression patterns of CHD1L in primary ovarian lesions and in metastatic leisions.** (**A**) Negative expression of CHD1L protein was observed in a primary ovarian lesions (case 75), in which less than 10% tumor cells revealed positive immunostaining of CHD1L in nuclei (*upper panel*, ×40). (**B**) A primary ovarian lesion case (case 45) demonstrated positive expression of CHD1L protein, in which more than 80% of tumor cells showed high immunoreactivity of CHD1L protein in nuclei (*upper panel*, ×40). (**C**) Positive expression of CHD1L protein was demonstrated in a metastatic ovarian lesion case (case 20, *upper panel*, ×40). The *lower panels* indicated the higher magnification (×200) from the area of the box in A, B and C respectively.

For CHD1L protein IHC staining in ovarian carcinoma tissues, positive staining was seen primarily in the nuclei within tumor cells, though occasionally yellowish brown granules could be also observed in the cytoplasm (Figure 1). The median score of CHD1L protein expression in the primary ovarian lesions is 4, while the score in the metastasis is 7. The difference between the levels of CHD1L expression in the primary lesions and in the metastatic lesions was statistically significant (*P <*0.05, Table 2).

**Table 2:** The expression of CHD1L protein in ovarian primary lesions and corresponding metastatic lesions

**Variable**	**CHD1L protein expression score**	**Z value**	*P *value*
ovarian primary lesions	4	−2.419	0.016
corresponding metastatic lesions	7		

* Wilcoxon Signed Ranks Test, *P*<0.05.

### Association of CHD1L protein expression with clinic-pathological parameters

In this study, according to the staining index above, protein expression with a scoring index of ≥ 4 (median score of CHD1L protein expression in the primary ovarian lesions) is defined as positive expression. The associations between CHD1L expression in primary ovarian carcinomas and several clinico-pathological variables are assessed and displayed in Table 1. The positive expression of CHD1L protein expression increasingly presented from mucinous/serous ovarian carcinoma to others types of tumor, including undifferentiated ovarian carcinoma. There was no significant difference between CHD1L protein expression and other clinicopathological features, such as patients’ age, histological grade, FIGO stage and residual tumor (*P*>0.05, Table 1).

### Relationship between clinicpathologic variables, CHD1L protein expression, and ovarian carcinoma patient survival: univariate survival analysis

**Figure 2:**
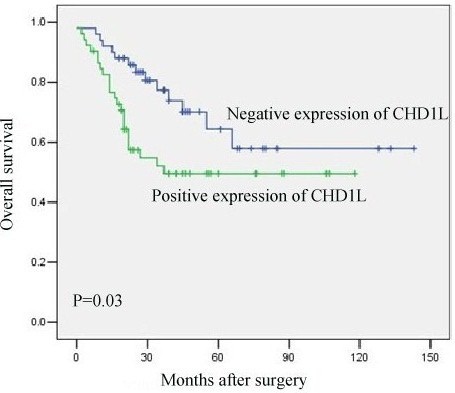
**Survival curve for 102 ovarian carcinoma patients according to CHD1L protein expression status (log-rank test).** Overall survival, probability of survival of all patients with ovarian carcinoma: negative expression, n=50; positive expression, n=52.

In univariate survival analysis, Kaplan-Meier survival curves and *P*-values for these curves were manipulated by log-rank method. Kapla-Meier analysis demonstrated a significant impact of well-known clinicopathological prognostic features such as histological grade, FIGO stage (*P*< 0.05, Table 3) and residual tumor (*P*< 0.05, Table 3). A statistically impaired overall survival was observed in patients with CHD1L-positive compared to patients with CHD1L-negative tumors. Mean overall survival time was 97.4 months for patients with CHD1L-negative expression compared to only 66.7 months for patients with CHD1L-positive expression (*P*< 0.05, Table 3, Figure 2).

**Table 3:** Clinical pathogical parameters and expression of CHD1L for prognosis of 102 patients with ovarian carcinoma by univariate survival analysis (log-rank test)

**Variable**	**All cases**	**Mean survival (months)**	**Median survival (months)**	***P *value**
Age at surgery				0.75
≤ 51^a^	54	74.4	NR^b^	
> 51	48	92.9	NR^b^	
Histological type				
Serous	72	78.4	NR^b^	0.55
Mucinous	11	62.7	45	
Others^c^	19	88.4	NR^b^	
Histologicalgrade (Silveberg)				0.00
G1	16	116.3	NR^b^	
G2	61	87.5	NR^b^	
G3	25	33.3	22	
FIGO stage				0.00
I	19	57.2	NR^b^	
II	10	59.1	NR^b^	
III	58	34.1	NR^b^	
IV	15	21.6	NR^b^	
Operation				0.00
Satisfactory cytoreductive operation	35	92.3	NR^b^	
Stage operation	32	121.4	NR^b^	
Unsatisfactory cytoreductive operation	25	47.6	22	
Chemical program				0.17
TP	17	62.0	NR^b^	
Cyclophosphamide + Adriamycin + Carbo	41	83.5	66	
Others	44	63.6	NR^b^	
Residual tumor				0.00
None	32	121.2	NR^b^	
<=2cm	35	92.3	NR^b^	
>2cm	35	46.5	22	
CHD1L protein expression				0.03
Negative	50	97.4	NR^b^	
Positive	52	66.7	37	

^a^ Mean age.

^b^ Not reach.

^c^Endometrioid, Clear cell and Undifferentiated types.

### Independent prognostic factors of epithelial ovarian carcinoma: multivariate cox regression analysis

A multivariate statistic analysis based on the Cox proportional hazard model was used to test the independent prognostic value of each clinicopathological feature (Table 4). Positive expression of CHD1L protein, as well as other clinicopathological variables (FIGO stage and residual tumor) which were significant by univariate analysis, was included in multivariate analysis. The CHD1L protein was discovered to be an independent prognostic factor for poor overall survival (*P*< 0.01, Table 4).

**Table 4:** Multivariate analysis on overall survival (Cox regression model)

**Variable**	**Relative risk**	**95% confidence interval**	***P *value**
CHD1L^a^	1.864	2.600-15.992	0.000
FIGO stage^b^	1.099	1.395 - 6.456	0.005
residual tumor^c^	0.926	1.253 - 5.089	0.010

^a^ Positive expressin vs Negative expression.

^b^Stage I vs Stage II vs Stage III vs StageIV.

^c^0cm vs <=2cm vs >2cm.

### Relationship between clinicpathologic variables, CHD1L protein expression, and ovarian carcinoma patient survival: receiver operating characteristic curve (ROC) analysis

**Figure 3:**
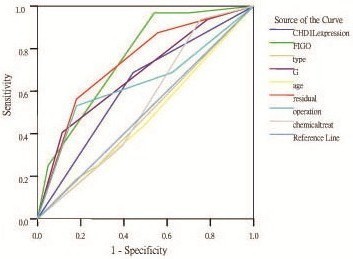
**ROC curve analysis for different clinicopathological parameters and CHD1L protein expression was performed to evaluate the survival status.** FIGO stage (area under the curve [AUC] =0.755, *P*<0.001), CHD1L protein expression ([AUC] =0.622, *P*<0.05), Residual tumor ([AUC] =0.737, *P*<0.001), Pathological grade ([AUC] =0.681, *P<*0.001) implied significant statistical associations with the survival.

The ROC for each clinicopathological parameter clearly reveal the point on the curve closest to (0.0, 1.0) which maximizes both sensitivity and specificity for the outcome. The ROC analysis for each clinicopathological variables and CHD1L expression (area under the curve [AUC] =0.622, *P*=0.05) is carried out to evaluate the patient’s survival status (Figure 3).

## Discussion

CHD1L, the candidate oncogene, has been isolated from 1q21 amplicon and found frequently amplified in hepatocellular carcinoma (HCC). Amplification of 1q21 is the most frequent genetic alteration in human HCC [14]. It plays an important role in the development and metastasis of HCC. Gains of 1q21-q25 have been linked to a more aggressive phenotype with metastatic potential in renal clear cell carcinoma and squamous cell carcinomas of lung [15,16]. In ovarian carcinoma and neuroblastoma, 1q21-q22 amplification indicated a drug-resistant phenotype [17,18]. CHD1L is activated by poly (ADP-ribose) (PAR) polymerase-1 (PARP-1), a DNA nick sensor enzyme that is activated by DNA breaks, during the posttranslational modification process termed “PARylation” [19]. CHD1L normally loosens tightly packaged chromatin to allow for repairing of DNA damage. In cancer cells, however, too much CHD1L can overly relax the chromatin, making the DNA vulnerable to mistake and thus raising the risk of cancer. However, to date, the expression dynamics of CHD1L in ovarian carcinomas has not been investigated, and its clinico-pathological and/or prognostic value in ovarian carcinomas remains unknown.

**Figure 4:**
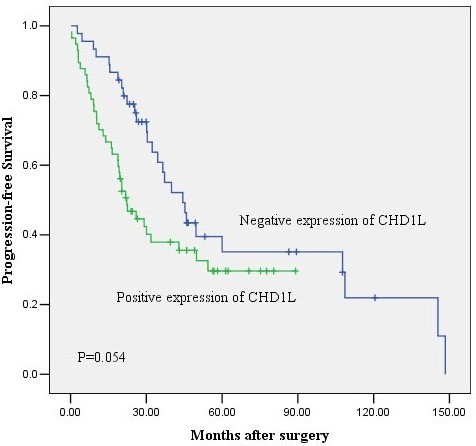
**Kaplan – Meier progression-free survival curves for the negative expression group(n=45) and the positive expression group(n=57).** The difference in progression-free survival rate between the two groups was significant.

In the present study, IHC staining for CHD1L was performed in a large cohort of ovarian carcinoma patients with complete clinicopathological and follow-up data. Our results clearly showed that the levels of CHD1L protein presented ascending in the metastatic lesions of ovarian carcinoma than that in the primary lesions, although no significant difference of CHD1L expression was evaluated between primary ovarian carcinomas in different stages. These results are in line with our previous findings in HCCs, in which we compared the expression of CHD1L by IHC between primary HCCs and their paired metastatic tumors, using a tissue microarray containing 50 pairs of primary and metastatic HCCs, and found that overexpression of CHD1L was a key feature in tumor development and metastasis. Increased expression of CHD1L was often observed at the invasive front of HCC tumors and correlated with venous infiltration, microsatellite tumor nodule formation [8]. These data, taken together, support the hypothesis that CHD1L might play an important role in the metastatic nature of several types of human cancer, such as HCC and ovarian carcinoma, and CHD1L protein overexpression in ovarian carcinoma may facilitate cancer cell metastasis. Further analysis demonstrated that the protein expression of CHD1L was only found to be correlated with histotypes, but was not found to be correlated with some significant impact of well-known clinicopathologic prognostic features such as histological grade, FIGO stage. However, in univariate analysis of the present study, we observed that the mean survival time for patients with CHD1L protein negative expression was 97.4 months compared to 66.7 months for patients with CHD1L protein positive expression( *P*<0.05). These findings was also similar to that in a cohort of HCC patients of our previous study, in which the median overall survival times in CHD1L-positive HCC patients is shorter than in CHD1L-negative patients [12]. Furthermore, in our study, we discovered that positive expression of CHD1L was an independent predictor of shorter overall survival in ovarian carcinomas as evidenced by Kaplan-Meier curves and multivariable Cox proportional hazards regression analysis. In summary, we did find that CHD1L expression, as detected by IHC, was a reliable biomarker for the prognosis of ovarian carcinoma patients, these data supports that CHD1L might act as a prognostic biomarker for patients with ovarian carcinoma independent of clinicopathologic prognostic parameters. More over, comparison of the progression-free survival rates of the negative expression (n=45) and positive expression (n=57) groups, shown in Figure 4, revealed statistical significant difference in the progression-free survival rate between the two groups(*P*=0.054).Thus, the examination of CHD1L expression by IHC could be used as an additional tool in identifying those ovarian carcinoma patients at increased risk of tumor metastasis and/or having a worse prognosis. These findings imply that CHD1L may play an important role in ovarian carcinoma development.

With regards to the gene function and oncogenic role of CHD1L, it is still not thoroughly characterized and clarified so far. Our recent in vitro and in vivo functional studies in mice showed that CHD1L contributed to HCC cell aggressiveness by increasing cell motility and inducing filopodia formation and epithelial-mesenchymal transition (EMT) via ARHGEF9-mediated Cdc42 activation. Silencing ARHGEF9 expression by RNAi lagely abolished the invasive and metastatic abilities of CHD1L in mice [8]. Furthermore, our investigation of clinical HCC specimens showed that CHD1L and ARHGEF9 were markedly overexpressed in metastatic HCC tissue compared with healthy tissue [8]. These findings suggested that CHD1LARHGEF9-Cdc42-EMT might be a novel pathway involved in HCC progression and/or metastasis. However, the potential oncogenic role and underling mechanisms of CHD1L in ovarian carcinoma have not been investigated. Clearly, further studies are needed to elucidate the mechanisms by which CHD1L promoting the development and metastasis of ovarian carcinomas in detail.

## Conclusions

In summary, we have shown here that CHD1L protein expression was significantly higher in in metastatic lesions than in the primary lesions and that CHD1L expression was significantly correlated with survival. Therefore, CHD1L is not only a biomarker associated with ovarian cancer, but it also appears to be a prognostic factor associated with adverse outcome.

## Competing interests

The authors declare that they have no competing interests.

## Authors' contributions

WPH carried out the immunohistochemistry assays, evaluated the clinical records and drafted the manuscript. JZ helped to carry out the immunohistochemistry assays and draft the manuscript. MYC participated in the statistical analysis and participated in its coordination. XSX and YJL helped to carry out the immunohistochemistry assays. HFK, XYG and DX participated in the design of the study, in its analysis and in the interpretation of the data. GFY designed the study and also participated in evaluated the immunohistochemistry results and wrote the manuscript. All authors read and approved the final manuscript.

## Pre-publication history

The pre-publication history for this paper can be accessed here:

http://www.biomedcentral.com/1471-2407/12/437/prepub
